# Gap-Directed Translesion DNA Synthesis of an Abasic Site on Circular DNA Templates by a Human Replication Complex

**DOI:** 10.1371/journal.pone.0093908

**Published:** 2014-04-07

**Authors:** Giuseppe Villani, Igor Shevelev, Eleonora Orlando, Helmut Pospiech, Juhani E. Syvaoja, Enni Markkanen, Ulrich Hubscher, Nicolas Tanguy Le Gac

**Affiliations:** 1 Centre National de la Recherche Scientifique, Institut de Pharmacologie et de Biologie Structurale, Toulouse, France; 2 Université de Toulouse, Université Paul Sabatier, Institut de Pharmacologie et de Biologie Structurale, Toulouse, France; 3 Leibniz Institute for Age Research - Fritz Lipman Institute, Jena, Germany; 4 Department of Biochemistry, University of Oulu, Oulu, Finland; 5 Institute of Biomedicine, University of Eastern Finland, Kuopio, Finland; 6 Biochemistry Group, Department of Oncology, Gray Institute for Radiation Oncology and Biology, Oxford, United Kingdom; 7 Institute of Veterinary Biochemistry and Molecular Biology, University of Zürich-Irchel, Zürich, Switzerland; Institute of Molecular Genetics IMG-CNR, Italy

## Abstract

DNA polymerase ε (pol ε) is believed to be the leading strand replicase in eukaryotes whereas pols λ and β are thought to be mainly involved in re-synthesis steps of DNA repair. DNA elongation by the human pol ε is halted by an abasic site (apurinic/apyrimidinic (AP) site). We have previously reported that human pols λ, β and η can perform translesion synthesis (TLS) of an AP site in the presence of pol ε. In the case of pol λ and β, this TLS requires the presence of a gap downstream from the product synthetized by the ε replicase. However, since these studies were conducted exclusively with a linear DNA template, we decided to test whether the structure of the template could influence the capacity of the pols ε, λ, β and η to perform TLS of an AP site. Therefore, we have investigated the replication of damaged “minicircle” DNA templates. In addition, replication of circular DNA requires, beyond DNA pols, the processivity clamp PCNA, the clamp loader replication factor C (RFC), and the accessory proteins replication protein A (RPA). Finally we have compared the capacity of unmodified versus monoubiquitinated PCNA in sustaining TLS by pols λ and η on a circular template. Our results indicate that *in vitro* gap-directed TLS synthesis by pols λ and β in the presence of pol ε, RPA and PCNA is unaffected by the structure of the DNA template. Moreover, monoubiquitination of PCNA does not affect TLS by pol λ while it appears to slightly stimulate TLS by pol η.

## Introduction

At least three DNA polymerases (pols) are required for chromosomal DNA replication in eukaryotic cells: pol α, pol δ and pol ε. Pol α has an associated primase activity necessary for initiation of replication [1], [2]; subsequently processive DNA synthesis is resumed by pol δ and ε. Data from yeast indicate that pol ε primarily replicates the DNA leading strand and pol δ the lagging strand [3].

Abasic sites (AP sites) arise frequently by spontaneous hydrolysis of purines in DNA and are a common intermediate of numerous DNA repair systems. AP sites are among the most frequent endogenous DNA lesions [4], [5] and pose a serious problem to the advancement of pols because the modified bases have lost their coding capacity. Very recently, an additional primase/polymerase, named PrimPol has been identified in Human cells [6]–[8] and whether this enzyme is required for unperturbed chromosomal DNA replication or following DNA damage or in both cases is still a matter of debate.

Translesion synthesis (TLS) of an AP site can be accomplished *in vitro* by either Y or X family pols [9]–[11]. It has also been reported that an AP site can be bypassed *in vitro* by pol α [12], by pol δ in the presence of the processivity clamp PCNA [13] and by PrimPol [6]–[8]. In contrast, elongation by human pol ε appeared to be blocked mainly at the base preceding the lesion, with minor incorporation opposite to it [14].

A widely accepted model of TLS is the polymerase-switching model, in which protein-protein interaction leads to a switch between the replicative pol arrested at a lesion and a pol capable of bypass. In eukaryotes, this switching appears to be mediated by a monoubiquitinated form of PCNA, although monoubiquitination appears to be important but not essential for TLS [10], [15].

Conversely, another model, named gap-filling model, can be taken in consideration. This model could apply to TLS in DNA gaps resulting from re-priming events or processing of closely spaced lesions on opposite DNA strands [10]. In contrast to the polymerase-switching model, the possible molecular mechanisms underlying the gap-filling model remain largely unknown.

Recently we have presented evidence that human pols λ, β and η can perform TLS of an AP site in the presence of pol ε, likely by extending the 3′OHs created at the lesion by the arrested pol ε [16]. Pols λ and β require for this TLS the presence of a DNA gap downstream from the product synthesized by pol ε, and the optimal gap length for efficient TLS is different for the two pols. Collectively, these results support the existence of a mechanism for gap-directed TLS at an AP site involving a switch between the replicative pol ε and the repair pols λ and β.

However, since these studies were conducted exclusively with a linear DNA template, we decided to examine whether the structure of the template could influence the capacity of the pols ε, λ and β to perform TLS of an AP site. This was investigated by testing replication of damaged “minicircle” DNA templates. These circular templates-primers were engineered so that their replication by pol ε led to the formation of single–stranded gaps of different length downstream the AP site. Replication of circular DNA requires, beyond DNA pols, the processivity clamp PCNA, the clamp loader replication factor C (RFC), and the accessory proteins replication protein A (RPA). We have also compared the capacity of unmodified versus monoubiquitinated PCNA in sustaining TLS by pols λ, β and η.

Our results indicate that *in vitro* gap-directed TLS synthesis by pols λ and β in the presence of pol ε, RPA, RFC and PCNA is unaffected by the structure of the DNA template and that monoubiquitination of PCNA does not influence TLS by pol λ while it appears to stimulate TLS by pol η.

## Materials and Methods

### Proteins

Recombinant human pol λ, RPA and PCNA were expressed and purified as described [17]–[19]. Site-specifically monoubiquitinated PCNA was synthesized as described [20]. Recombinant human pol β was from Trevigen Inc. (Gaithesburg, MD). Recombinant human pol η was from Enzymax (Lexington, KY). Human pol ε was purified from HeLa cells trough six purification steps as described [14]. The glycerol gradient fraction used in this study had a specific activity of 24,000 units/mg. Its purity was estimated to be >50% and the fraction was devoid of other replicative pols. Human recombinant pol δ was expressed and purified as described [21]. His–tagged human RFC subunits were expressed in Baculovirus. Cells were collected and lysed in buffer LEW (50 mM NaH_2_PO_4_ pH 8, 300 mM NaCl and protease inhibitor cocktail-Roche) and centrifuged at 20,000 rpm 1 hour in JA-20 rotor. The supernatant was mixed with Ni-DA beds in LEW buffer for 4 hours at 4°C and then poured into a column. Elution was performed with LEW plus 250 mM Imidazol. The 2 ml eluted fraction was diluted at 100 mM NaCl with buffer A (50 mM this pH 8, 50 mM NaCl, 15% glycerol, 1 mM EDTA, 1 mM 2-mercaptoethanol and 2 mM protease inhibitors) and charged on a mono Q column equilibrated with buffer A. Fractions of 0.4 ml were eluted with a gradient of 0.1 to 0.4 NaCl in buffer A and the protein peak fractions tested for the capacity to stimulate pol δ activity in presence of PCNA on a minicircular template (see Results and Discussion).

### DNA substrates and chemicals

**Figure 1:**
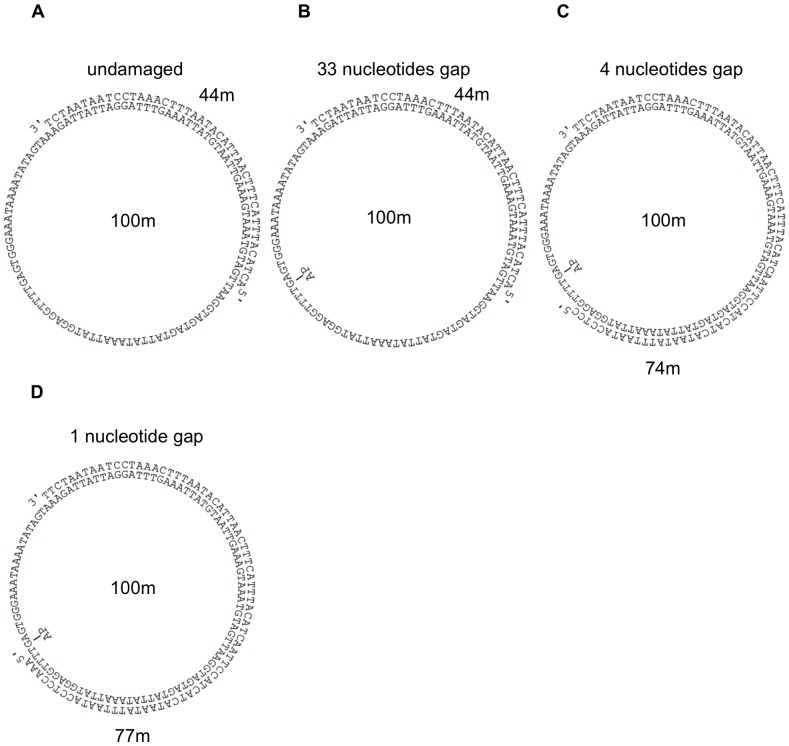
DNA templates –primers used in this study. AP indicates the position of the synthetic abasic site at the G position of the undamaged template. For details see Materials and Methods

The 100-mer oligonucleotide linear template, either undamaged or containing a synthetic AP site (tetrahydrofuran moiety) and the oligonucleotides used as scaffold for constructing circular DNA templates or as primers for extension assays, were from Eurogentec and were PAGE purified. For a detailed description of the method used to construct a mini-circular DNA template starting from a linear one see [22]. The DNA templates-primers used in this study are shown in Fig 1. Primers were 5′-labelled with T4 polynucleotide kinase (New England Biolabs) in the presence of [γ-^32^P]-ATP according to the manufacturer's protocol. Each primer was mixed with the templates at a 1∶1 (M/M) ratio in the presence of 20 mM Tris HCl pH 8 and 50 mM KCl, heated at 90°C for 5 minutes and then slowly cooled. [γ-^32^P]-ATP was from Perkin Elmer, dNTPs were from Fermentas, and 20× Glycerol Tolerant Gel (GTG) buffer was from USB.

### Primer extension assays

Reaction mixtures of 15 μl were incubated at 37°C and contained 0.15 pmoles of DNA templates, 50 mM Hepes KOH pH 7.5, 8 mM MgCl_2_, 1 mM DTT, 200 μg/ml BSA, 2.5% Glycerol, 1.5 mM ATP and 50 μM each of dATP, dCTP, dGTP and dTTP. The incubation times and the amounts or of proteins used are indicated in the legends of the figures. The reactions were stopped by adding 7.5 μl of stop solution containing 0.1% xylene cyanol and 0.1% bromophenol blue in 90% formamide. Before loading onto the gel, samples were denatured by heating at 100°C for 3 min. The reaction products were resolved on denaturing polyacrylamide gel electrophoresis (7 M urea, 10% acrylamide), run in GTG buffer (90 mM Tris HCL pH 9, 30 mM taurine and 5 mM EDTA), visualized and quantified using Molecular Dynamics PhosphorImager and ImageQuant software. The percentage of translesion synthesis (TLS) was calculated as the ratio of the intensity of bands present at the position opposite the lesion or beyond to the intensity of these bands plus the one present one nucleotide before the lesion.

## Results and Discussion

The structure of the DNA could affect the TLS activity of a DNA polymerase. Indeed, the *E. coli* replicase DNA polymerase III holoenzyme showed a limited capacity to replicate through an abasic site only when the lesion was present on a circular DNA substrate [23].

**Figure 2:**
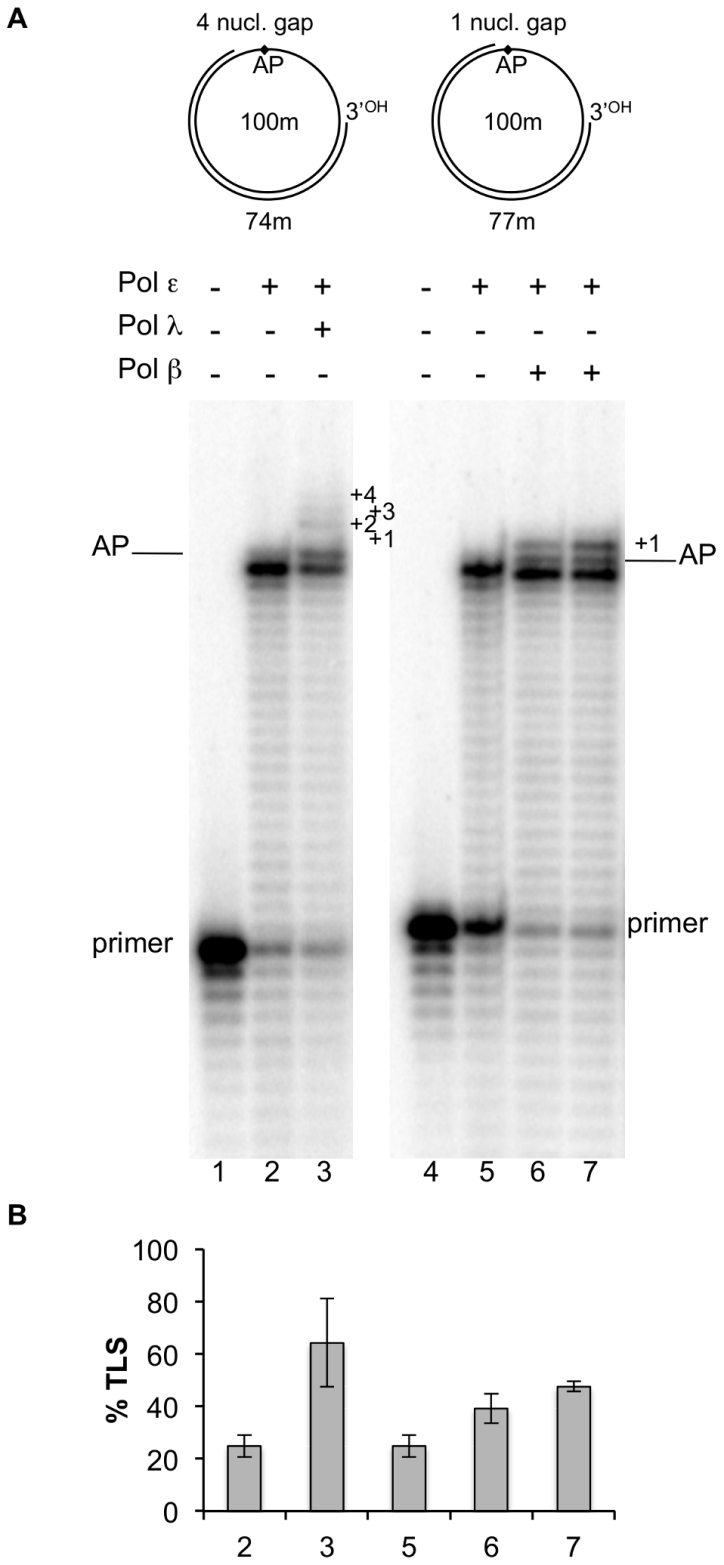
Translesion synthesis over an AP site by DNA Polymerases λ and β in the presence of DNA DNA polymerase ε on circular DNA templates with gaps of four or one nucleotides downstream the lesion. Experiments were performed with damaged templates with either 74 or 77 mer primers, as depicted on the top of Fig 2A and in Fig 1. The enzymes are indicated at the top of the Figure. Experiments were carried out as described in Materials and Methods. A: Lane 1, no polymerase present. Lane 2, reaction incubated for 35 minutes with 0.025 pmol of pol ε. Lane 3, reaction incubated for 30 minutes with 0.025 pmol of pol ε, then 0.25 pmol of pol λ was added and the incubation was continued for 5 minutes. Lane 4, no polymerase present. Lane 5, reaction incubated for 35 minutes with 0.025 pmol of pol ε. Lane 6, reaction incubated for 30 minutes with 0.025 pmol of pol ε, then 0.25 pmol of pol β was added and the incubation continued for 5 minutes. Lane 7, reaction incubated for 30 minutes with 0.025 pmol of pol ε, then 0.25 pmol of pol β was added and the incubation continued for 10 minutes. The position of the primers, the AP site and the nucleotides past the AP site are indicated. B: Quantification of the percentage of TLS, calculated as described in Materials and Methods. Mean +/− S.D. values for three independent experiments are indicated.

We have recently shown that human pols λ, β and η can perform TLS of an AP site in the presence of pol ε; this TLS required, for pols λ and β, the presence of a DNA gap downstream from the product synthesized up to the lesion by pol ε [16]. Furthermore, no TLS by pol λ and β was detected in the presence of a gap of a length longer than 13 nucleotides. Since this study was conducted on a linear DNA template-primer, we investigated whether these conclusions were also valid with a circular DNA. To permit a direct comparison with the data obtained with the linear DNA, we circularized the 100-mer template used in the previous study and we primed it with oligonucleotides of different length in order to create gaps of 33, 4 and 1 nucleotides respectively, downstream the position of the AP site following replication by pol ε up to the lesion (substrates B, C and D of Fig 1). As expected the enzyme stopped at the nucleotide preceding the lesion, with nearly 20% incorporation in front of it (lane 2 and 5 of Fig 2A, quantified in B). At this time point either pol λ or pol β were added. With the circular DNA bearing a 33 nucleotides gap we observed no TLS upon addition of pol λ or β. On the contrary, efficient TLS by pol λ and β was observed with a 4 or 1 nucleotide gap templates-primers respectively (lanes 3, 6 and 7 of Fig 2A, quantified in B). These data paralleled those obtained with the linear template and show that pol λ and β can performed TLS on a circular DNA in the presence of pol ε only if DNA gaps of appropriate lengths are present downstream the lesion.

Accessory replicative proteins such as the processivity clamp PCNA and the single-stranded DNA-binding protein RPA play a fundamental role in DNA replication, repair and recombination [2], [5].

PCNA is a ring shaped homotrimeric protein that is loaded on the primer-template junction by a multiprotein clamp loader, replication factor C (RFC), which couples the hydrolysis of ATP with the opening and closing of the PCNA ring around the DNA [24]. In the presence of RFC, PCNA increases the processivity of the replicative pol δ [2] but its capacity to stimulate or not the processivity of pol ε remains controversial, possibly depending on the type of DNA substrates and experimental conditions used [25], [26].

In this study we have used RFC (see Materials and Methods for its purification) and verified its functionality by monitoring its capacity to stimulate elongation by pol δ in the presence of PCNA on the undamaged minicircular template-primer A depicted in Fig 1.

**Figure 3:**
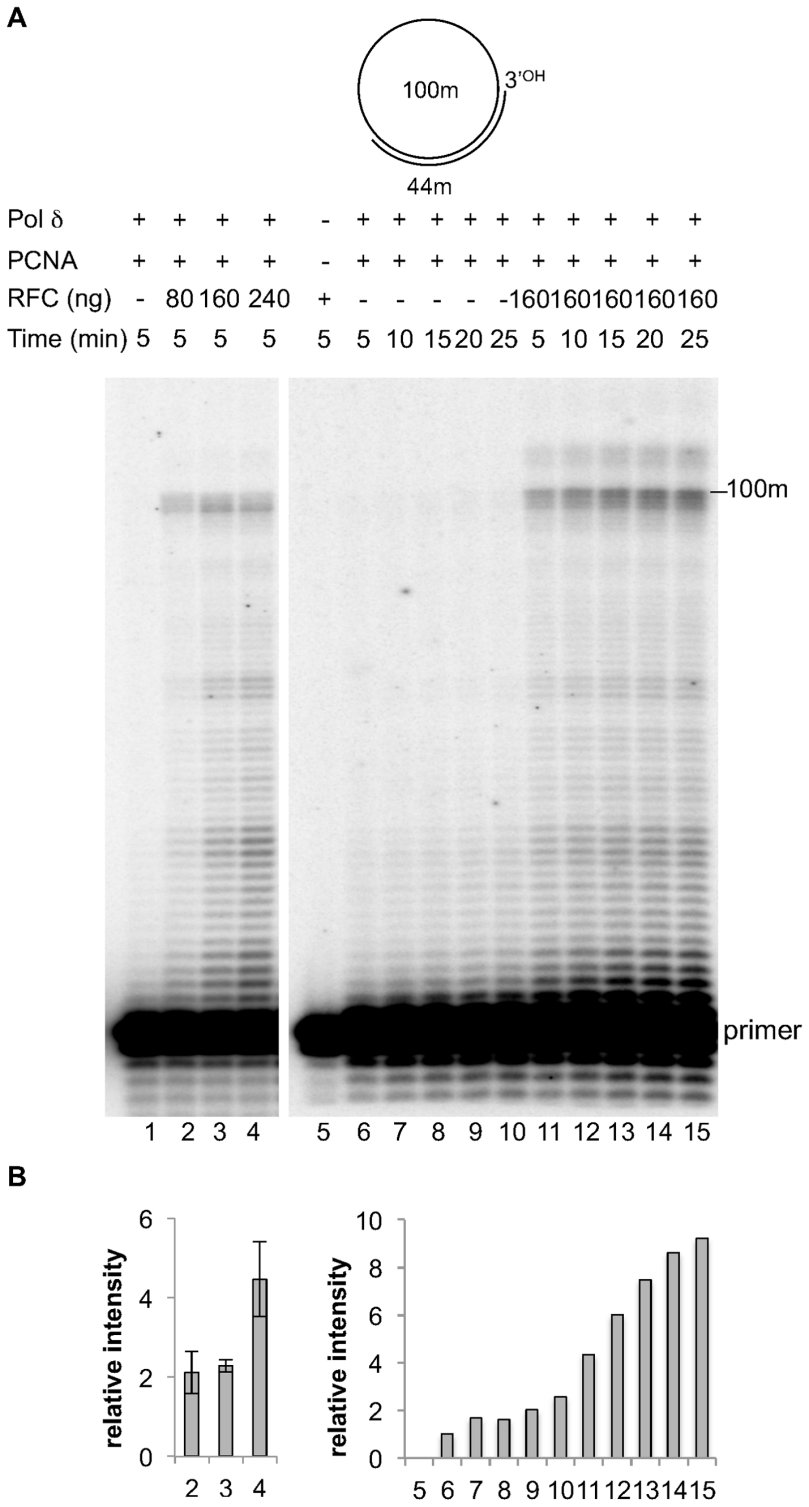
RFC stimulation of PCNA dependent synthesis on a circular DNA template. Experiments were performed with the intact template and a 44Fig 1. The proteins are indicated at the top of the Figure. Experiments were carried out as described in Materials and Methods. **A**: Lane 1, reaction incubated for 5 minutes with 0.03 pmol of pol δ and 1 pmol of PCNA. Lane 2, reaction incubated for 5 minutes with 0.03 pmol of pol δ, 1 pmol of PCNA and 80 ngs of RFC. Lane 3, reaction incubated for 5 minutes with 0.03 pmol of pol δ, 1 pmol of PCNA and 160 ngs of RFC. Lane 4, reaction incubated for 5 minutes with 0.03 pmol of pol δ, 1 pmol of PCNA and 240 ngs of RFC. Lane 5, 160 ngs of RFC alone incubated for 25 minutes. Lane 6–10, reactions incubated with 0.03 pmol of pol δ and 1 pmol of PCNA for 5,10,15,20 and 25 minutes respectively. Lane 11–15, reactions incubated with 0.03 pmol of pol δ, 1 pmol of PCNA and 160 ngs of RFC for 5,10,15,20 and 25 minutes respectively. **B**: Quantification of the data in **A** by plotting the relative intensity of the bands migrating higher than the primer. The positions of the primer and of the 100 mer full length product are indicated. Left graph shows mean +/− S.D. values for three independent experiments. Right graph shows the quantification of the right panel of figure 3A.

First, we checked the capacity of different concentrations of RFC to stimulate PCNA dependent pol δ synthesis. As it can be seen in Fig 3A lanes 1–4, quantified in Fig 3B, a robust stimulation was observed already at the first concentration of RFC used, and this effect increased with higher amounts of the protein.

We also performed a kinetic of stimulation and found that an increasing effect was observed up to 25 minutes of incubation (Fig 3A lanes 11–15, quantified in B). Moreover, some reaction products of higher molecular weight than the 100 mer full size replication product were also detected in the presence of RFC (lanes 7 to 11) which are likely to be the result of strand displacement synthesis by pol δ in presence of PCNA [27]. Note that 160 ngs RFC alone did not display any polymerase activity under our experimental conditions (lane 5 of Fig 3), and that no stimulation of pol δ was observed in the presence of RFC but in the absence of PCNA (data not shown).

Taken together, these data indicated that RFC substantially stimulated DNA synthesis by pol δ in the presence of PCNA, thus effectively acting as clamp loader under our experimental conditions.

**Figure 4:**
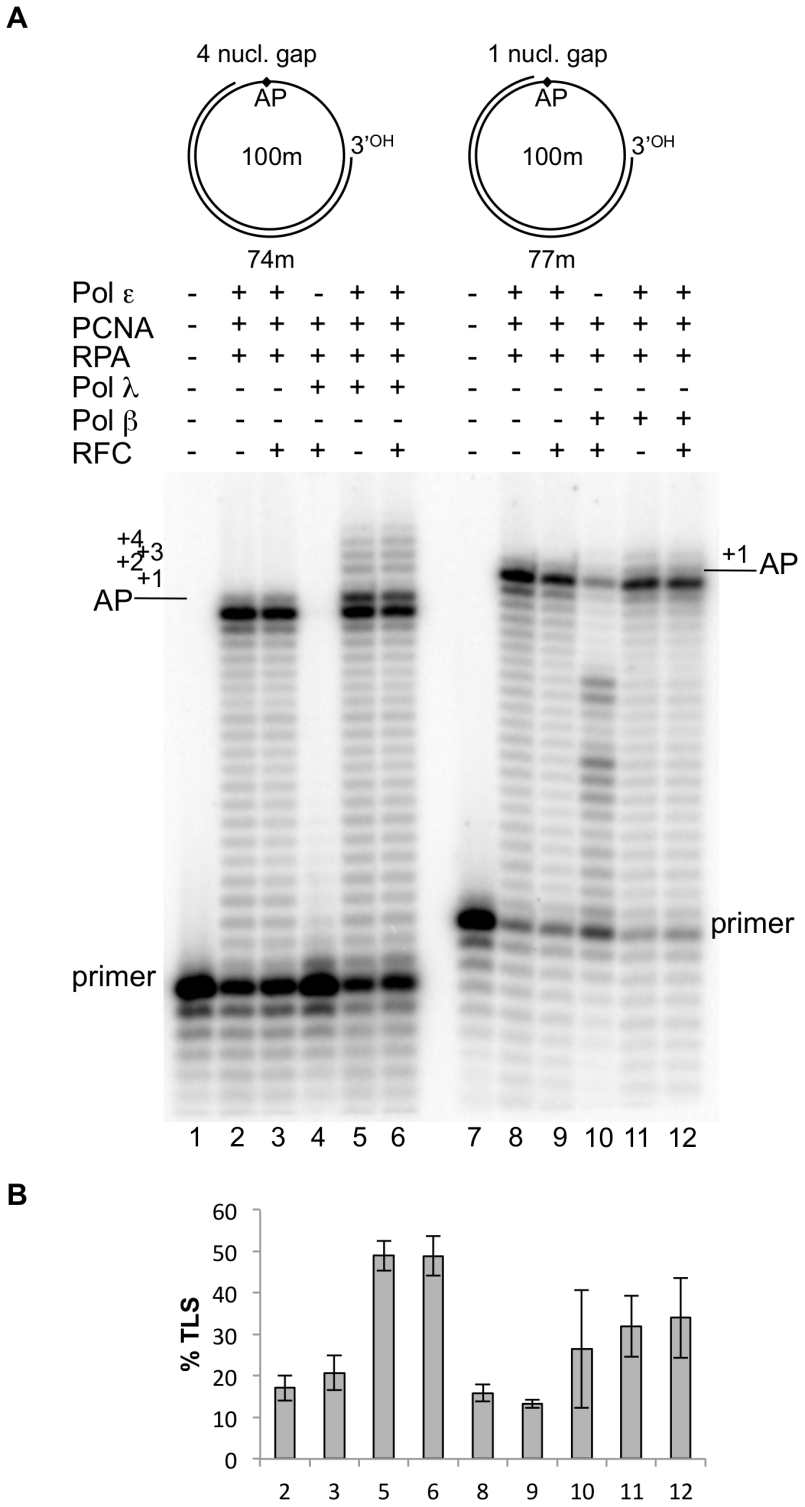
Translesion synthesis over an AP site on circular DNA templates by a reconstituted replication complex. Experiments were performed with damaged templates primed with either 74 or 77 mer primers, as depicted on the top of Fig 4A and in Fig 1. The proteins are indicated at the top of the Figure. Experiments were carried out as described in Materials and Methods. **A**: Lane 1, no proteins present. Lane 2, reaction incubated for 35 minutes with 0.025 pmol of pol ε, 1 pmol of PCNA and 0.25 pmol of RPA. Lane 3, reaction incubated for 35 minutes with 0.025 pmol of pol ε, 1 pmol of PCNA, 0.25 pmol of RPA and 160 ngs of RFC. Lane 4, reaction incubated 5 minutes with 0.25 pmol of pol λ, 1 pmol PCNA, 0.25 pmol RPA and 160 ngs of RFC. Lane 5, reaction incubated for 30 minutes with 0.025 pmol of pol ε, 1 pmol of PCNA and 0.25 pmol of RPA; then 0.25 pmol of pol λ was added and the incubation was continued for 5 minutes. Lane 6, reaction incubated for 30 minutes with 0.025 pmol of pol ε, 1 pmol of PCNA, 0.25 pmol of RPA and 160 ngs of RFC; then 0.25 pmol of pol λ was added and the incubation was continued for 5 minutes. Lane 7, no proteins present. Lane 8, reaction incubated for 35 minutes with 0.025 pmol of pol ε, 1 pmol of PCNA and 0.25 pmol of RPA. Lane 9, reaction incubated for 35 minutes with 0.025 pmol of pol ε, 1 pmol of PCNA, 0.25 pmol of RPA and 160 ngs of RFC. Lane 10, reaction incubated 5 minutes with 0.25 pmol of pol β, 1 pmol of PCNA, 0.25 pmol of RPA and 160 ngs RFC. Lane11, reaction incubated for 30 minutes with 0.025 pmol of pol ε, 1 pmol of PCNA and 0.25 pmol of RPA; then 0.25 pmol of pol β was added and the incubation was continued for 5 minutes. Lane 12, reaction incubated for 30 minutes with 0.025 pmol of pol ε, 1 pmol of PCNA, 0.25 pmol of RPA and 160 ngs of RFC; then 0.25 pmol of pol β was added and the incubation was continued for 5 minutes. The position of the primers, the AP site and the nucleotides past the AP site are indicated. **B**: quantification of the percentage of TLS, calculated as described under Materials and Methods. Mean +/− S.D. values for three independent experiments are indicated.

Next, we investigated whether the TLS catalysed at gaps in circular DNA by pols λ and β in presence of pol ε could be influenced by human PCNA, RPA and RFC. Previous data with linear DNA indicated that a 4 nucleotide gap is the optimal substrate to monitor TLS of the AP site by pol λ [16]. Therefore, the circular DNA bearing such a gap was chosen as template-primer for the experiments which are presented in the left part of Fig 4A. As it can be seen in lanes 2, pol ε, in presence of PCNA and RPA, was unable to replicate past the AP site and mainly stopped at the base preceding the lesion with some incorporation opposite it. Addition of RFC did not change the pattern of the reaction products (lane 3). Addition of pol λ led to TLS of the AP site to fill the 4 nucleotide gap downstream the lesion (lane 5). Addition of RFC did not change the TLS capacity (lane 6). When pol λ alone was incubated for 5 min with the substrate (lane 4), the products synthesized were too short to reach the lesion, suggesting that pol λ catalysed TLS by using the 3′OH created by pol ε. Similar results were obtained by pol β using the circular 1 nucleotide-gap substrate that in its linear form was found to be optimal for TLS by pol β [16] and are presented in the right part of Fig. 4A. As it can be seen (lanes 8 to 12), addition of PCNA, RPA or RFC did not change the DNA synthesis pattern of pol ε alone or the TLS capacity of pol β in the presence of pol ε.

Fig 4B quantifies the data shown in Fig. 4A. As it can be seen, under the same experimental conditions, the extent of TLS detected by pols λ and β is highly comparable to the one observed in the absence of PCNA, RPA and RFC (see lanes 3 and 6 of Fig 2B). These results strongly indicate that TLS by the two pols is not influenced by these accessory factors when replicating a circular DNA template.

**Figure 5:**
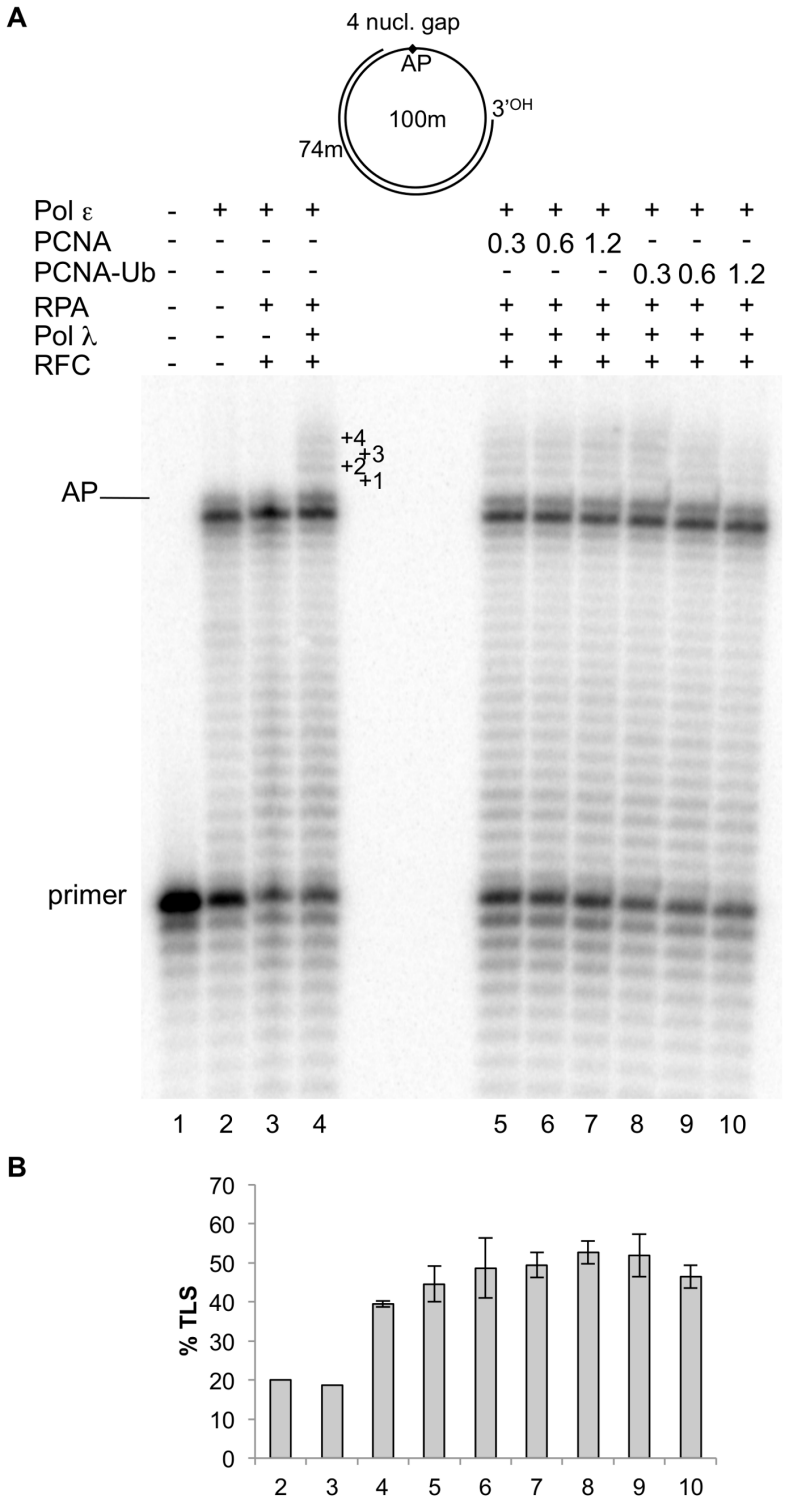
Monoubiquitination of PCNA does not change the pattern of TLS by DNA polymerase λ. Experiments were performed with a damaged template primed with the 74 mer primer, as depicted on the top of the Fig 5A and in Fig 1. The proteins are indicated at the top of the figure. Experiments were carried out as described in Materials and Methods. **A**: lane 1, no proteins present. Lane 2, reaction incubated for 35 minutes with 0.025 pmol of pol ε. Lane 3, reaction incubated for 35 minutes with 0.025 pmol of pol ε, 0.25 pmol of RPA and 160 ngs of RFC. Lane 4, reaction incubated for 30 minutes with 0.025 pmol of pol ε, 0.25 pmol of RPA and 160 ngs of RFC; then 0.25 pmol of pol λ was added and the incubation continued for 5 minutes. Lanes 5 to 7; as in lane 4 but with 0.3, 0.6 and 1.2 pmol of PCNA respectively. Lanes 8 to 10; as in lane 4 but with 0.3, 0.6 and 1.2 pmol of monoubiquitinated PCNA respectively. The position of the primers, the AP site and the nucleotides past the AP site are indicated. **B**: Quantification of the percentage of TLS, calculated as described in Materials and Methods. Mean +/− S.D. values for three independent experiments are indicated.

Monoubiquitination of PCNA at lys 164 (PCNA-Ub) appears to be necessary to make it capable of stimulating TLS by translesion pols in the cell [2]. Therefore we investigated whether monoubiquitination of PCNA could affect gap directed TLS of an AP site by pol λ in the presence of pol ε, RPA and RFC. We directly compared TLS by this polymerase on the 4 nucleotides gapped circular DNA when increasing amount of either unmodified or monoubiquitinated PCNA were present. The result of such experiment is shown in Fig. 5A and quantified in 5B. As expected, efficient TLS by pol λ is achieved already in absence of PCNA (lane 4). As it can be seen, addition of either increasing amount of unmodified or monoubiquitinated PCNA did not affect the extent of the TLS reaction (compare lanes 5–7 to lanes 8–10), indicating that monoubiquitination of PCNA does not play a significant role in the TLS of gapped circular DNA by pol λ.

**Figure 6:**
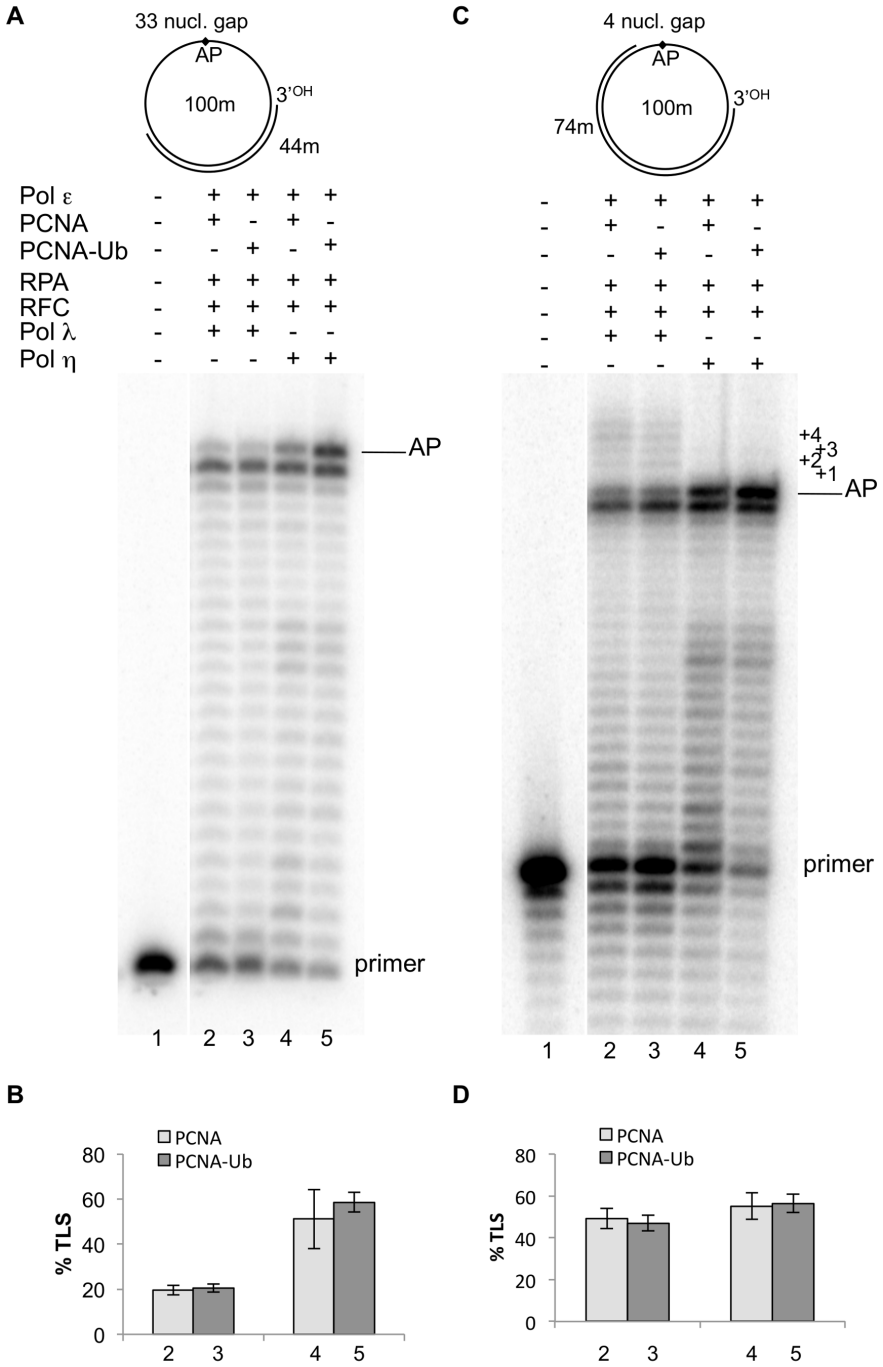
Monoubiquitinated PCNA stimulates TLS by DNA polymerase η. Experiments were performed with damaged templates primed with the 44 or 74 mer primers, as depicted on the top of Fig 6A and in Fig 1. The proteins are indicated at the top of the Fig. Experiments were carried out as described in Materials and Methods. **A**: 1, no proteins present. Lane2, reaction incubated for 30 minutes with 0.025 pmol of pol ε, 1.2 pmol of PCNA, 0.25 pmol of RPA and 160 ngs of RFC; then 0.25 pmol of pol λ was added and the incubation continued for 5 minutes. Lane 3, as in lane 2 but with 1.2 pmol of monoubiquinated PCNA. Lane 4, reaction incubated for 30 minutes with 0.025 pmol of pol ε, 1.2 pmol of PCNA, 0.25 pmol of RPA and 160 ngs of RFC; then 0.25 pmol of pol η was added and the incubation continued for 5 minutes. Lane 5, as in lane 4 but with 1.2 pmol of monoubiquinated PCNA. The positions of the primer and of the AP site are indicated. **B**: quantification of the percentage of TLS of the data shown in **A**, calculated as described in Materials and Methods. Error bars reflect ranges from two independent experiments. **C**: 1, no proteins present. Lane2, reaction incubated for 30 minutes with 0.025 pmol of pol ε, 1.2 pmol of PCNA, 0.25 pmol of RPA and 160 ngs of RFC; then 0.25 pmol of pol λ was added and the incubation was continued for 5 minutes. Lane 3, as in lane 2 but with 1.2 pmol of monoubiquinated PCNA. Lane 4, reaction incubated for 30 minutes with 0.025 pmol of pol ε, 1.2 pmol of PCNA, 0.25 pmol of RPA and 160 ngs of RFC; then 0.25 pmol of pol η was added and the incubation was continued for 5 minutes. Lane 5, as in lane 4 but with 1.2 pmol of monoubiquinated PCNA. The positions of the primer and of the AP site are indicated. **D**: quantification of the percentage of TLS of the data shown in **C**, calculated as described in Materials and Methods. Error bars reflect ranges from two independent experiments.

Interaction of the translesion pol η with monoubiquitinated PCNA has been suggested to mediate the polymerase switch at a lesion [28]. However, the role of PCNA-Ub on *in vitro* TLS of an AP site by pol η remains controversial [29], [30]. We have previously shown that, in the presence of pol ε, pol η could perform TLS of an AP site on a linear DNA template, mainly by increasing incorporation in front of the lesion [16]; however this TLS could take place also on a 33 nucleotides large gap where TLS by pols λ and β was not detected. Therefore we assayed the capacity of pol λ and pol η to perform TLS on a circular DNA template in the presence of pol ε, the accessory replicative proteins RPA, RFC and either unmodified or monoubiquitinated PCNA (Fig 6).

First we examined the replication of an AP site placed on a template bearing a gap of 33 nucleotides downstream the lesion. As expected, pol λ could not perform TLS on such template with either unmodified or PCNA-Ub (lanes 3 and 4 of Fig 6A, quantified in B). At variance with this, in the presence of PCNA, pol η showed an increased capacity to incorporate in front of the AP site, and this capacity appeared to be slightly higher in the presence of PCNA-Ub (lanes 4 and 5 of Fig 6A, quantified in B).

Next the TLS capacity of the two enzymes at an AP site placed on a template bearing a gap of 4 nucleotides downstream the lesion was tested. Pol λ could perform TLS in the presence of pol ε and accessory proteins that resulted in filling of the gap with the same efficiency either with unmodified PCNA or PCNA-Ub (lanes 2 and 3 of Fig 6C quantified in D). Pol η mainly catalysed increased incorporation in front of the AP site, that seemed slightly higher with PCNA-Ub, but could not fill the 4 nucleotides gap (lanes 4 and 5 Fig 6C, quantified in D).

Taken together these results suggest that, under our experimental conditions, pol η has the capacity to incorporate in front of an AP site in the presence of pol ε and accessory replicative proteins. However, this capacity, differently from the case of pol λ, is not gap directed and appears to be moderately enhanced by PCNA-Ub when a gap of 33 nucleotides is present.

In conclusion, we show here that human pols λ and β can efficiently replicate, in the presence of pol ε, RPA and PCNA, an AP site placed in a gap situated downstream the lesion in a circular DNA template. These data are in agreement with those already reported for linear DNA templates containing an AP site [16] or benzopyrene adducts [31], and indicate that the mechanism (s) leading to this TLS are unaffected by the structure of the DNA templates. In this work we also add novel findings suggesting that the presence of RFC and monoubiquitinated PCNA does not influence the TLS by pol λ while it appears to stimulate TLS by pol η.

Taken together, these results indicate that the known high affinity for gap binding of pols λ and β is the major if not unique biochemical determinant responsible for their gap-directed bypass capacity of lesions in the template strand.

## References

[1] Kornberg A, Baker TA (2005) DNA Replication. 2nd ed. University Science Books.

[2] Hübscher U, Spadari S, Villani G, Maga G (2010) DNA Polymerases: Discovery, Characterization and Functions in Cellular DNA Transactions. 1st ed. World Scientific Publishing Company.

[3] Kunkel TA, Burgers PM (2008) Dividing the workload at a eukaryotic replication fork. Trends Cell Biol 18: 521–527. 18824354 10.1016/j.tcb.2008.08.005 PMC2665207

[4] Lindahl T (1993) Instability and decay of the primary structure of DNA. Nature 362: 709–715. 8469282 10.1038/362709a0

[5] Friedberg EC, Walker GC, Siede W, Wood RD, Schultz RA, et al.. (2005) DNA Repair And Mutagenesis. 2nd ed. ASM Press.

[6] García-Gómez S, Reyes A, Martínez-Jiménez MI, Chocrón ES, Mourón S, et al (2013) PrimPol, an Archaic Primase/Polymerase Operating in Human Cells. Molecular Cell 52: 541–553. 24207056 10.1016/j.molcel.2013.09.025 PMC3899013

[7] Bianchi J, Rudd SG, Jozwiakowski SK, Bailey LJ, Soura V, et al (2013) PrimPol Bypasses UV Photoproducts during Eukaryotic Chromosomal DNA Replication. Molecular Cell 52: 566–573. 24267451 10.1016/j.molcel.2013.10.035 PMC4228047

[8] Wan L, Lou J, Xia Y, Su B, Liu T, et al (2013) hPrimpol1/CCDC111 is a human DNA primase-polymerase required for the maintenance of genome integrity. EMBO Rep 14: 1104–1112. 24126761 10.1038/embor.2013.159 PMC3981091

[9] Prakash S, Johnson RE, Prakash L (2005) Eukaryotic translesion synthesis DNA polymerases: specificity of structure and function. Annu Rev Biochem 74: 317–353. 15952890 10.1146/annurev.biochem.74.082803.133250

[10] Waters LS, Minesinger BK, Wiltrout ME, D'Souza S, Woodruff RV, et al (2009) Eukaryotic translesion polymerases and their roles and regulation in DNA damage tolerance. Microbiol Mol Biol Rev 73: 134–154. 19258535 10.1128/MMBR.00034-08 PMC2650891

[11] Blanca G, Villani G, Shevelev I, Ramadan K, Spadari S, et al (2004) Human DNA polymerases lambda and beta show different efficiencies of translesion DNA synthesis past abasic sites and alternative mechanisms for frameshift generation. Biochemistry 43: 11605–11615. 15350147 10.1021/bi049050x

[12] Shibutani S, Takeshita M, Grollman AP (1997) Translesional synthesis on DNA templates containing a single abasic site. A mechanistic study of the “A rule”. J Biol Chem 272: 13916–13922. 9153253 10.1074/jbc.272.21.13916

[13] Mozzherin DJ, Shibutani S, Tan CK, Downey KM, Fisher PA (1997) Proliferating cell nuclear antigen promotes DNA synthesis past template lesions by mammalian DNA polymerase delta. Proc Natl Acad Sci USA 94: 6126–6131. 9177181 10.1073/pnas.94.12.6126 PMC21013

[14] Locatelli GA, Pospiech H, Tanguy Le Gac N, van Loon B, Hübscher U, et al (2010) Effect of 8-oxoguanine and abasic site DNA lesions on in vitro elongation by human DNA polymerase in the presence of replication protein A and proliferating-cell nuclear antigen. Biochem J 429: 573–582. 20528769 10.1042/BJ20100405

[15] Hendel A, Krijger PHL, Diamant N, Goren Z, Langerak P, et al (2011) PCNA ubiquitination is important, but not essential for translesion DNA synthesis in mammalian cells. PLoS Genetics PLoS Genet 7(9): e1002262 doi:10.1371/journal.pgen.1002262 21931560 10.1371/journal.pgen.1002262 PMC3169526

[16] Villani G, Hübscher U, Gironis N, Parkkinen S, Pospiech H, et al (2011) In vitro gap-directed translesion DNA synthesis of an abasic site involving human DNA polymerases epsilon, lambda, and beta. J Biol Chem 286: 32094–32104. 21757740 10.1074/jbc.M111.246611 PMC3173188

[17] Shevelev I, Blanca G, Villani G, Ramadan K, Spadari S, et al (2003) Mutagenesis of human DNA polymerase lambda: essential roles of Tyr505 and Phe506 for both DNA polymerase and terminal transferase activities. Nucleic Acids Res 31: 6916–6925. 14627824 10.1093/nar/gkg896 PMC290264

[18] Henricksen LA, Umbricht CB, Wold MS (1994) Recombinant replication protein A: expression, complex formation, and functional characterization. J Biol Chem 269: 11121–11132. 8157639

[19] Jónsson ZO, Hindges R, Hübscher U (1998) Regulation of DNA replication and repair proteins through interaction with the front side of proliferating cell nuclear antigen. The EMBO Journal 17: 2412–2425. 9545252 10.1093/emboj/17.8.2412 PMC1170584

[20] Eger S, Castrec B, Hübscher U, Scheffner M, Rubini M, et al (2011) Generation of a mono-ubiquitinated PCNA mimic by click chemistry. Chembiochem 12: 2807–2812. 22052741 10.1002/cbic.201100444

[21] van Loon B, Ferrari E, Hübscher U (2009) Isolation of recombinant DNA elongation proteins. Methods Mol Biol 521: 345–359. 19563116 10.1007/978-1-60327-815-7_19

[22] Tanguy Le Gac N, Delagoutte E, Germain M, Villani G (2004) Inactivation of the 3“-5” exonuclease of the replicative T4 DNA polymerase allows translesion DNA synthesis at an abasic site. J Mol Biol 336: 1023–1034. 15037066 10.1016/j.jmb.2004.01.005

[23] Tomer G, Livneh Z (1999) Analysis of unassisted translesion replication by the DNA polymerase III holoenzyme. Biochemistry 38: 5948–5958. 10231549 10.1021/bi982599+

[24] Indiani C, O'Donnell M (2006) The replication clamp-loading machine at work in the three domains of life. Nat Rev Mol Cell Biol 7: 751–761. 16955075 10.1038/nrm2022

[25] Syvaoja J, Suomensaari S, Nishida C, Goldsmith JS, Chui GS, et al (1990) DNA polymerases α, δ, and ε: three distinct enzymes from HeLa cells. Proc Natl Acad Sci USA 87: 6664–6668. 1975694 10.1073/pnas.87.17.6664 PMC54597

[26] Kanuri M, Minko IG, Nechev LV, Harris TM, Harris CM, et al (2002) Error prone translesion synthesis past gamma-hydroxypropano deoxyguanosine, the primary acrolein-derived adduct in mammalian cells. J Biol Chem 277: 18257–18265. 11889127 10.1074/jbc.M112419200

[27] Maga G, Villani G, Tillement V, Stucki M, Locatelli GA, et al (2001) Okazaki fragment processing: modulation of the strand displacement activity of DNA polymerase delta by the concerted action of replication protein A, proliferating cell nuclear antigen, and flap endonuclease-1. Proc Natl Acad Sci USA 98: 14298–14303. 11724925 10.1073/pnas.251193198 PMC64676

[28] Kannouche PL, Wing J, Lehmann AR (2004) Interaction of human DNA polymerase eta with monoubiquitinated PCNA: a possible mechanism for the polymerase switch in response to DNA damage. Molecular Cell 14: 491–500. 15149598 10.1016/s1097-2765(04)00259-x

[29] Garg P, Burgers PM (2005) Ubiquitinated proliferating cell nuclear antigen activates translesion DNA polymerases eta and REV1. Proc Natl Acad Sci USA 102: 18361–18366. 16344468 10.1073/pnas.0505949102 PMC1317920

[30] Haracska L, Unk I, Prakash L, Prakash S (2006) Ubiquitylation of yeast proliferating cell nuclear antigen and its implications for translesion DNA synthesis. Proc Natl Acad Sci USA 103: 6477–6482. 16611731 10.1073/pnas.0510924103 PMC1458909

[31] Chary P, Beard WA, Wilson SH, Lloyd RS (2012) DNA polymerase β gap-filling translesion DNA synthesis. Chem Res Toxicol 25: 2744–2754. 23121263 10.1021/tx300368f PMC3523550

